# Evaluating the glucose raising effect of established loci via a genetic risk score

**DOI:** 10.1371/journal.pone.0186669

**Published:** 2017-11-10

**Authors:** Eirini Marouli, Stavroula Kanoni, Vasiliki Mamakou, Sophie Hackinger, Lorraine Southam, Bram Prins, Angela Rentari, Maria Dimitriou, Eleni Zengini, Fragiskos Gonidakis, Genovefa Kolovou, Vassilis Kontaxakis, Loukianos Rallidis, Nikolaos Tentolouris, Anastasia Thanopoulou, Klea Lamnissou, George Dedoussis, Eleftheria Zeggini, Panagiotis Deloukas

**Affiliations:** 1 William Harvey Research Institute, Barts and The London School of Medicine and Dentistry, Queen Mary University of London, United Kingdom; 2 Dromokaiteio Psychiatric Hospital, Athens, Greece; 3 Wellcome Trust Sanger Institute, Hinxton, Cambridge; 4 Wellcome Trust Centre for Human Genetics, Oxford, United Kingdom; 5 Department of Nutrition and Dietetics, School of Health Science and Education, Harokopio University, Athens, Greece; 6 Department of Oncology and Metabolism, University of Sheffield, Sheffield, United Kingdom; 7 1st Psychiatric Department, National and Kapodistrian University of Athens, Medical School, Eginition Hospital, Athens, Greece; 8 Department of Cardiology, Onassis Cardiac Surgery Center, Athens, Greece; 9 Early Psychosis Unit, 1st Department of Psychiatry, National and Kapodistrian University of Athens, Medical School, Eginition Hospital, Athens, Greece; 10 Second Department of Cardiology, National and Kapodistrian University of Athens, Medical School, Attikon Hospital, Athens, Greece; 11 First Department of Propaedeutic and Internal Medicine, National and Kapodistrian University of Athens, Medical School, Laiko General Hospital, Athens, Greece; 12 Diabetes Centre, 2nd Department of Internal Medicine, National and Kapodistrian University of Athens, Medical School, Hippokration General Hospital, Athens, Greece; 13 Division of Genetics & Biotechnology, Department of Biology, National & Kapodistrian University of Athens, Greece; 14 Princess Al-Jawhara Al-Brahim Centre of Excellence in Research of Hereditary Disorders (PACER-HD), King Abdulaziz University, Jeddah, Saudi Arabia; South Texas Veterans Health Care System, UNITED STATES

## Abstract

Recent genome-wide association studies have identified several single nucleotide polymorphisms (SNPs) associated with glucose levels. We tested the hypothesis here whether the cumulative effect of glucose raising SNPs, assessed via a score, is associated with glucose levels. A total of 1,434 participants of Greek descent from the THISEAS study and 1,160 participants form the GOMAP study were included in this analysis. We developed a genetic risk score (GRS), based on the known glucose-raising loci, in order to investigate the cumulative effect of known glucose loci on glucose levels. In the THISEAS study, the GRS score was significantly associated with increased glucose levels (mmol/L) (β ± SE: 0.024 ± 0.004, P = 8.27e-07). The effect of the genetic risk score was also significant in the GOMAP study (β ± SE: 0.011 ± 0.005, P = 0.031). In the meta-analysis of the two studies both scores were significantly associated with higher glucose levels GRS: β ± SE: 0.019 ± 0.003, P = 1.41e-09. Also, variants at the *SLC30A8*, *PROX1*, *MTNR1B*, *ADRA2A*, *G6PC2*, *LPIN3* loci indicated nominal evidence for association with glucose levels (*p* < 0.05). We replicate associations of the established glucose raising variants in the Greek population and confirm directional consistency of effects (binomial sign test p = 6.96e-05). We also demonstrate that the cumulative effect of the established glucose loci yielded a significant association with increasing glucose levels.

## Introduction

Type 2 diabetes is a metabolic disorder resulting in altered protein, carbohydrate and fat metabolism and is characterized by impaired insulin secretion, impaired glucose and insulin homeostasis. Pre-diabetic abnormalities, such as impaired fasting glucose and impaired glucose tolerance could lead to diabetes [1–3]. A large number of genome-wide significant loci influencing glycemic traits [4] and type 2 diabetes development [5, 6] have been recently identified. The results from these studies highlight important biological pathways implicated in glucose regulation [7, 8]. The number of common associated SNPs with fasting glucose concentration has now raised to 36[9]. Many of these loci include those encoding genes involved in b-cell function, insulin secretion and some are encoding transcription factors implicated in pancreas development[4]. A number of the loci associated with glycemic traits in diabetes-free individuals also affect the risk of type 2 diabetes [10]. More specifically, thirty three out of 53 glycemic loci are also associated with increased type 2 diabetes risk [4]. The fact that the observed effect sizes are generally small lends further support that a significant number of variants with small effect size is more likely to explain a substantial proportion of the variance in glucose levels [11, 12]. The medical socioeconomic strain of the disease is increased by its complications [13]. Identification of a large number of common and rare genetic variants implicated in glucose metabolism contributes to translate the genetic information to the clinical practice and improve risk prediction.

Genetic risk models for type 2 diabetes based on both cross-sectional and longitudinal studies have been developed [14, 15]. The little observed clinical value of genetic testing for identification of individuals prone to develop impaired glucose metabolism can be improved in the future with new sequencing techniques and identification of low and rare variants with larger effect sizes[14]. Evaluation of the cumulative variant effect of glucose risk loci on glucose levels could give a good estimation for pre-diabetic status as well as better control and prevention. This is of particular interest as based on recent estimations from IDF, 415 million people have diabetes in the world and there were 608,800 cases of diabetes in Greece in 2015 (http://www.idf.org/membership/eur/greece). Over the last 30 years, the number of diabetic individuals has at least quadrupled. It is imperative to focus our research on ways that could detect individuals at risk of developing diabetes, by identifying them when they are in the pre-diabetic or borderline state. If undiagnosed or untreated, prediabetes can develop into type 2 diabetes, which is not fully reversible and is a global problem closely tied to obesity.

Here we address the role of the association of known glucose-raising genetic variants with glucose levels. We sought to evaluate the cumulative effect of the known variants on glucose levels estimated by the calculation of an unweighted and a weighted genetic risk score in the Greek population.

## Materials and methods

### Study population

Our sample consisted of 2594 individuals from Greek origin individuals, aged 57.1 ± 13.6 years old. The sample was drawn from the THISEAS (The Hellenic Study of Interactions between SNPs and Eating in Atherosclerosis Susceptibility) [16, 17] and GOMAP (Genetic Overlap of Metabolic and Psychiatric Diseases) studies. 1434 individuals were included from the THISEAS and 1160 individuals for the GOMAP study, all diabetes-free adult individuals of Greek ancestry (S1 Table). Association analyses were undertaken in individuals deriving from each study separately. Meta-analysis was carried out in the GOMAP and THISEAS studies. All participants were informed both verbally and in a written consent form [16]. The studies had the approval of the Ethics Committee of Harokopio University of Athens.

### Adiposity and biochemical measurements

The enzymatic colorimetric assay (ACE analyzer) was used for glucose levels measurements in THISEAS and GOMAP studies. Venus serum was used for glucose levels. Individuals with type 2 diabetes were excluded based on their medical history; self-reported cases in THISEAS. We also excluded individuals with fasting glucose levels more than 7 mmol/L and random glucose levels more than 11 mmol/L.

### Genotyping

Genomic DNA was extracted from whole blood using the iPrep PureLink gDNA Blood Kit-Invitrogen for the GOMAP study and by using the salting-out method for the THISEAS study[18]. DNA samples were genotyped using the Illumina Metabochip for the THISEAS study and using the Illumina HumanCoreExomeChip at the Wellcome Trust Sanger Institute, Hinxton, UK. The genotype calling algorithms were GenoSNP and GenCall for THISEAS and GOMAP studies respectively. For both studies we excluded individuals with sample call rate < 95%, genome-wide heterozygosity higher than ± 3SD, sex discrepancies, ethnic outliers identified by multidimensional scaling (MDS) using PLINK[19]. In THISEAS study four SNPs (rs2657879, rs2302593, rs7708285 and rs10830963) failed the 98% call rate threshold and were replaced by imputed data. Imputation was performed using the cosmopolitan 1000 Genomes panel (http://www.1000genomes.org/) and IMPUTE2 [20]. Prior phasing was done using SHAPEIT [21]. In the GOMAP study twelve SNPs failed the 98% call rate threshold and were replaced by imputed data. Imputation was performed using a 3-way reference panel (1000Genomes (http://www.1000genomes.org/), UK10K (http://www.uk10k.org/) and MANOLIS study). Estimated probabilities for these SNPs were converted to best-guess genotypes using gtool (http://www.well.ox.ac.uk/~cfreeman/software/gwas/gtool.html). In the imputed format each SNP is represented as a set of three probabilities which corresponds to allele pairs AA, AB and BB. Using a calling threshold cutoff for the probability values (info: 0.9), probabilities for each individual were transformed to best-guess genotypes using Gtool (http://www.well.ox.ac.uk/~cfreeman/software/gwas/gtool.html). The genotypes are expressed as pairs of 1, 2, 0 where 1 corresponds to allele A from GEN file and 2 corresponds to allele B. If none of the probabilities are over the calling threshold then the pair is unknown, coded as 0 0. The defined threshold for calling genotypes (info: 0.9) was used in order to calculate for missing data. The info measure takes the value 1 if all genotypes are completely certain, and the value 0 if the genotype probabilities for each sample are completely uncertain.

### Genetic risk score (GRS) modelling

In order to investigate the cumulative effects of known glucose-raising genetic variants, a genetic risk score was constructed (GRS). We included the public available variants associated with glucose levels, identified by the MAGIC (the Meta-Analyses of Glucose and Insulin-related traits Consortium) effort [4, 22]. We calculated the GRS for each individual by summing the number of effect allele of the 36 SNPs they are carrying. We also modelled a weighted genetic risk score. The wGRS was calculated as the sum of the 36 risk alleles, weighted by the published effect sizes [4]. We further addressed the possible overfitting issue, as THISEAS study was part of the discovery effort, where the weights for the wGRS were taken from. We removed the effect of this particular sample, by using mathematical formulas to simulate a situation where this sample is excluded from the meta-analysis, and obtained corrected beta estimates [23]. For the calculation of the weighted scores in THISEAS study, only corrected effect sizes were used. We present the range of the possible number of weighted glucose-increasing alleles, by dividing the score by the average effect size of the 36 SNPs for each individual [24]. This is a transformation of the wGRS so that the range equalled that of the unweighted score.

### Statistical analysis

We used PLINK [19] assuming an additive genetic model to investigate the association of the 36 SNPs on glucose levels in both studies. We conducted an inverse variance weighted meta-analysis in a sample of individuals of Greek ancestry. GWAMA (Genome-Wide Association Meta-Analysis) was used for the meta-analysis performed for the two studies [25]. Statistical analysis was performed using R version 3.1.1. We present continuous variables as mean ± standard deviation (SD). The coefficient of determination (R^2^) was used to express the proportion of total variation explained by the GRS and the wGRS. We used t-test to compare the differences between two means.

Regression coefficients derived from the linear regression models testing for the association of the scores with glucose levels, express the increase of glucose levels per 1-unit increase in the genetic score. An inverse variance weighted meta-analysis for the association of the scores with glucose levels was performed for the two studies. The reported p values were based on two-sided tests and for the associations assessed adjustments for age, sex and BMI were applied. For the association analyses the statistical significance level is set at p≤0.05.

We used Quanto v1.2.4, for power calculations (http://hydra.usc.edu/gxe/). We had 80% power to detect a 0.045 mmol/L change in glucose levels under the effect of the genetic risk score (S1 Fig).

## Results

Several of the variants corresponding to previously identified MAGIC loci also showed evidence for association with glucose levels in our meta-analysis with adjustments used by large-scale meta-analyses for glycemic traits[26]. Nominal associations were observed at the *SLC30A8* (β ± SE: 0.053 ± 0.021, p = 0.013), *PROX1* (β±SE: 0.043±0019, p = 0.027), *MTNR1B* (β±SE: 0.048±022, p = 0.03), *ADRA2A* (β±SE: 0.074±035, p = 0.034), *G6PC2* (β±SE: 0.046±022, p = 0.035) and *LPIN3* (β±SE: 0.058±0027, p = 0.035) loci, after adjusting for age and sex (S2, S4 and S6 Tables, S4 Fig). The majority of the investigated variants (30 out of 36) had consistent direction of effect between the meta-analysis of the THISEAS and GOMAP studies and the MAGIC [4] meta-analysis (binomial sign test p = 6.96e-05). Nominally significant associations were observed after adjusting for age, sex and BMI for *MTNR1B*, *ADRA2A*, *SLC30A8*, *PDX1*, *FEN1*, *G6PC2* and *CDKAL1* loci (S3, S5 and S7 Tables).

**Fig 1:**
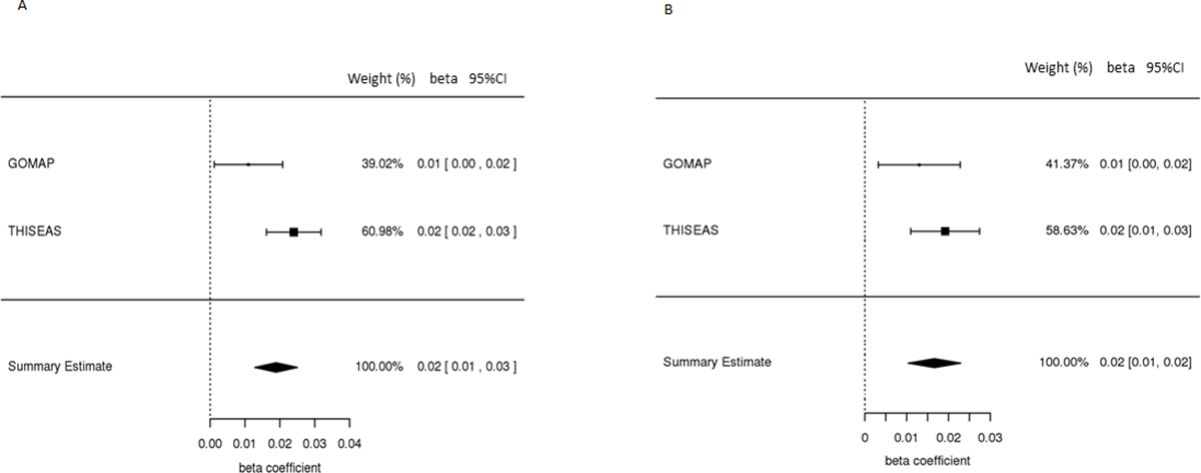
Forest plots of the Meta-Analysis results. Forest plots showing the beta estimates and confidence intervals of the A. GRS and B. wGRS association in the different populations studied in the discovery cohorts and the meta-analysis fixed model results. An inverse variance weighted meta-analysis was performed.

Association of the GRS and wGRS scores with glucose levels were also investigated. We observed positive correlation of the GRS and wGRS with glucose levels (mmol/L) in diabetes-free individuals for the THISEAS and GOMAP studies (S2 and S3 Figs). Both scores were significantly associated with higher glucose levels, as expected. For each increasing risk allele in an individual’s GRS, glucose levels were increased by 0.019 mmol/L: β ± SE: 0.019 ± 0.003, P = 1.41e^-9^. For each increment point in an individual’s wGRS, glucose levels were increased by 0.016 mmol/L: β ± SE: 0.016 ± 0.003, P = 3.75e^-8^ (Tables 1 and 2). Our results indicate no substantial difference regarding the increasing effect of the two scores reflected by the beta value. Forest plots reporting the effect size of the GRS and wGRS in both cohorts as well as in the meta-analysis are shown in Fig 1.

**Table 1:** Associations of the unweighted Genetic Risk Score (GRS) and the weighted Genetic Risk Score (wGRS) with glucose levels for the meta-analysis of the GOMAP and THISEAS studies ^a^.

Scores	β	SE	beta_95L	beta_95U	z	p-value	i2	N
**GRS36**	0.018	0.004	0.011	0.025	4.910	9.29E-07	0.624	2216
**wGRS36**	0.016	0.003	0.010	0.021	5.480	4.35E-08	0.000	2216

^a^Adjusted for age and sex

Beta coefficient and standard error for the estimated difference in glucose (mmol/L per 1-unit increase in the scores GRS and wGRS.

i2: Heterogeneity index I2 by Higgins *et al*. 2003.

N: indicates the sample size. The slight difference in sample size is due to missing BMI values in the studies.

**Table 2:** Associations of the unweighted Genetic Risk Score (GRS) and the weighted Genetic Risk Score (wGRS) with glucose levels for the meta-analysis of the GOMAP and THISEAS studies ^b^.

Scores	β	SE	beta_95L	beta_95U	z	p-value	i2	N
**GRS36**	0.019	0.003	0.013	0.025	6.060	1.41E-09	0.042	2160
**wGRS36**	0.016	0.003	0.010	0.022	5.506	3.75E-08	0.125	2160

^b^Adjusted for age, sex and BMI.

Beta coefficient and standard error for the estimated difference in glucose (mmol/L per 1-unit increase in the scores GRS and wGRS.

i2: Heterogeneity index I2 by Higgins *et al*. 2003.

N: indicates the sample size. The slight difference in sample size is due to missing BMI values in the studies.

**Fig 2:**
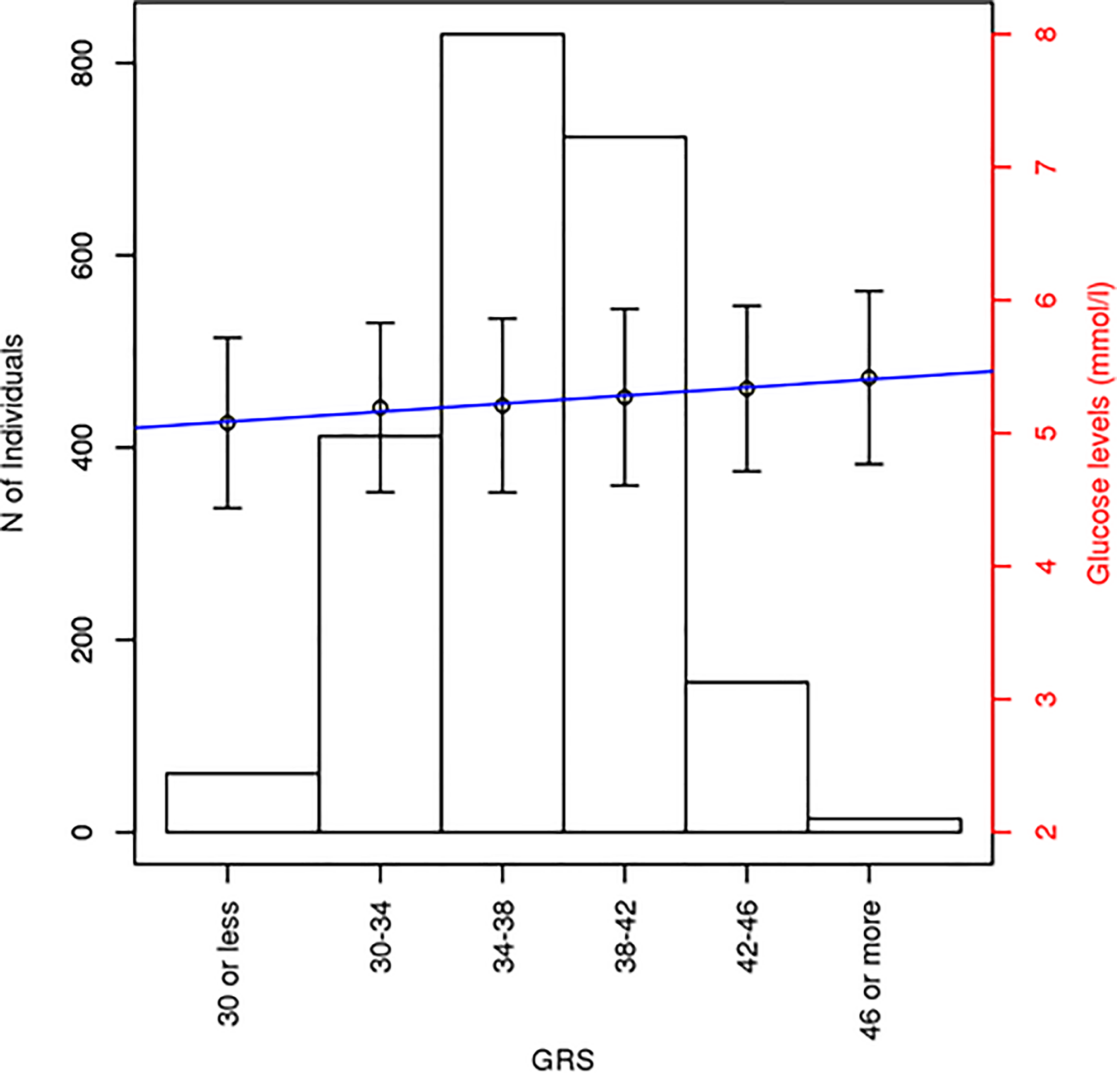
Distribution of individuals in every genotype score group and cumulative effects of the risk alleles from the 36 SNPs for glucose levels. The bar plots show the average and standard error of glucose in mmol/L for each genotype score group distribution of the genetic risk score.

The difference in mean glucose levels between subjects in the highest tertile and those in the lower tertile was 0.12191 mmol/L, P = 2.30 x10^-3^). Linear regression models for glucose levels estimation suggested the GRS account for 6.35% of the variance in glucose levels (Fig 2).

Descriptive characteristics of the study cohorts are given in S1 Table. The mean GRS and wGRS are 37.347 ± 3.686 and 38.729 ± 4.388 points respectively for the diabetes-free individuals from the THISEAS study and 37.651 ± 3.708 and 39.214 ± 4.134 points respectively for the diabetes-free individuals from the GOMAP study.

## Discussion

We constructed unweighted and weighted genetic risk scores based on 36 known variants associated with glucose levels [4]. Our purpose was to investigate whether these genetic scores are associated with increased glucose levels and could predict impaired glucose metabolism in a sample of the Greek population.

Many of the previously published variants associated with glucose levels also showed evidence of association in the present meta-analysis, fact that supports the investigation of their cumulative effect on glucose levels by using a genetic risk score in our population.

Given that most common risk variants identified until now have modest effects on glucose levels, we ought to investigate the cumulative effect of these variants on glucose levels. We conducted meta-analysis of the genetic scores using data from diabetes-free individuals from the THISEAS and GOMAP studies. The derived GRS and wGRS scores were associated with increased glucose levels.

There are some limitations that should be mentioned. The established variants associated with increased glucose levels used for the scores, explain only a small proportion of the variance in glucose levels. The observed lower beta estimate of the GRS for the GOMAP study could be attributed to the hospital-based recruitment, opposite to the THISEAS study, where volunteers were recruited from the general population. Furthermore, another limitation of this study is that individuals from the THISEAS study were included in the discovery meta-analysis of Scott *et al*., which identified 20 out of the 36, but not all glucose associated variants, which were used for the generation of the genetic risk score presented in the current manuscript [27]. To further address this issue, we performed the analyses for the wGRS in THISEAS study, using corrected estimates [23]. Also, observational studies, especially cross-sectional study designs are characterized by limited ability to offer causality or of predictive value inferences. Furthermore, gene-gene and gene-environment interactions should be taken into account in order to increase the glucose levels estimation ability of a genetic risk score.

In summary we found that both GRS and wGRS based on 36 well-established risk loci were significantly associated with glucose levels. We observed that the allele frequency of the established variants in the Greek population is in accordance with what is reported by MAGIC for the European populations. To this end it is generally anticipated that ongoing imputation and next-generation sequencing-based studies will identify further variants affecting blood glucose levels and increase the estimated variation in glucose levels attributed to genetics. It is possible that addition of a larger number of common and low frequency variants could improve risk prediction.

Genetic Risk Score methods when generalized could provide improved risk assessment and lead to personalized preventive care. Studying the genetic risk scores for common diseases in the general population is not straight-forward. It is also expected that genotypes individually have small effects for a complex diseases, such as diabetes. Investigating the drug response based on genotypes and stratifying individuals based on their genetic background could yield in a more effective treatment. By identifying individuals at risk of developing diabetes based on their GRS, clinicians could more accurately assess a patient’s unique risk of developing diabetes and therefore recommend personalized screening methods. In the era of personalized medicine incorporation of genetic risk scores could potentially have important clinical utility.

In total, a genetic risk score method could categorize individuals based on their clinical risk, by using many variants deriving from multiple genes and include them in a simpler model. The genetic scores created could be promising for improving risk stratification. Application of screening individuals for predisposing genetic variants, reliably associated with glycaemic traits, could contribute to better recommendations for glucose homeostasis control.

## Supporting information

S1 Fig Estimated power of the analyses for alpha 0.05. Lines represent the different power values to detect the effect in the model for the association of the weighted genetic risk score (GRS) with the trait. Calculations were performed using Quanto v1.2.4.
(TIF)

S2 Fig Scatterplot of the positive correlation between the genetic risk scores and glucose levels (mmol/L) in the GOMAP study. A. unweighted genetic risk score (GRS) and glucose levels (mmol/L) and B. weighted genetic risk score (wGRS) and glucose levels (mmol/L).
(TIF)

S3 Fig Scatterplot of the positive correlation between the genetic risk scores and glucose levels (mmol/L) in the THISEAS study. A. unweighted genetic risk score (GRS) and glucose levels (mmol/L) and B. weighted genetic risk score (wGRS) and glucose levels (mmol/L).
(TIF)

S4 Fig Meta-analysis association results for glucose levels associated loci with glucose levels (mmol/L). Adjusted for age and sex, x-axis: Gene name, y-axis: -logPvalue.
(TIF)

S1 Table Study-specific descriptive statistics of the cohorts. Age, weighted Genetic Risk Score (wGRS), unweighted Genetic Risk Score (GRS) and Glucose (mmol/L). Data are presented as means ± SD for the total sample.
(XLSX)

S2 Table THISEAS association summary statistics for glucose levels associated loci with glucose levels. Effect sizes (beta) and SE are given for Glucose (mmol/L), Imputation quality: info > 0.97.
Chr = chromosome, bp = base pairs, EAF = effect allele frequency, SE = standard error.
Allelic test p, beta and SE are shown for each single SNP.
Effect sizes (beta) are reported for the effect allele.
Results were obtained using linear regression models assuming an additive effect. Adjustments: age and sex.
EAF: Effect Allele Frequency, HWE: Hardy Weinberg Equilibrium.
(XLSX)

S3 Table THISEAS association summary statistics for glucose levels associated loci with glucose levels. Effect sizes (beta) and SE are given for Glucose (mmol/L), Imputation quality: info > 0.97.
Chr = chromosome, bp = base pairs, EAF = effect allele frequency, SE = standard error.
Allelic test p, beta and SE are shown for each single SNP.
Effect sizes (beta) are reported for the effect allele.
Results were obtained using linear regression models assuming an additive effect. Adjustments: age, sex and BMI.
EAF: Effect Allele Frequency, HWE: Hardy Weinberg Equilibrium.
(XLSX)

S4 Table GOMAP association summary statistics for glucose levels associated loci with glucose levels. Effect sizes (beta) and SE are given for Glucose (mmol/L), Imputation quality: info > 0.97.
Chr = chromosome, bp = base pairs, EAF = effect allele frequency, SE = standard error.
Allelic test p, beta and SE are shown for each single SNP.
Effect sizes (beta) are reported for the effect allele.
Results were obtained using linear regression models assuming an additive effect. Adjustments: age and sex.
EAF: Effect Allele Frequency, HWE: Hardy Weinberg Equilibrium.
(XLSX)

S5 Table GOMAP association summary statistics for glucose levels associated loci with glucose levels. Effect sizes (beta) and SE are given for Glucose (mmol/L), Imputation quality: info > 0.97.
Chr = chromosome, bp = base pairs, EAF = effect allele frequency, SE = standard error.
Allelic test p, beta and SE are shown for each single SNP.
Effect sizes (beta) are reported for the effect allele.
Results were obtained using linear regression models assuming an additive effect. Adjustments: age, sex and BMI.
EAF: Effect Allele Frequency, HWE: Hardy Weinberg Equilibrium.
(XLSX)

S6 Table Meta-analysis association results for glucose levels associated loci with glucose levels. Effect sizes (β) and se are given for Glucose (mmol/L).
Allelic test p, be and se are shown for each single SNP.
Effect sizes (β) are reported for the effect allele.
Adjustments: age and sex.
An inverse variance weighted meta-analysis was performed.
(XLSX)

S7 Table Meta-analysis association results for glucose levels associated loci with glucose levels. Effect sizes (β) and se are given for Glucose (mmol/L).
Allelic test p, be and se are shown for each single SNP.
Effect sizes (β) are reported for the effect allele.
Adjustments: age, sex and BMI.
An inverse variance weighted meta-analysis was performed.
(XLSX)

S8 Table Associations^a^ analyses of the Genetic Risk Scores with glucose levels. ^a^Adjusted for age and sex.
^b^Beta coefficient and standard error for the estimated difference in glucose (mmol/L) values per 1-unit increase in the genetic risk scores (GRS and wGRS respectively -A and B-).
^b^N indicates the sample size.
(XLSX)

S9 Table Associations^a^ analyses of the Genetic Risk Scores with glucose levels. ^a^Adjusted for age, sex and BMI.
^b^Beta coefficient and standard error for the estimated difference in glucose (mmol/L) values per 1-unit increase in the genetic risk scores (GRS and wGRS respectively -A and B-).
^b^N indicates the sample size.
(XLSX)
