# Supplementary figures and images for: Evaluating the glucose raising effect of established loci via a genetic risk score
Source: PLoS One. 2017 Nov 10;12(11):e0186669. doi: 10.1371/journal.pone.0186669 (PMC5681259; doi:10.1371/journal.pone.0186669)

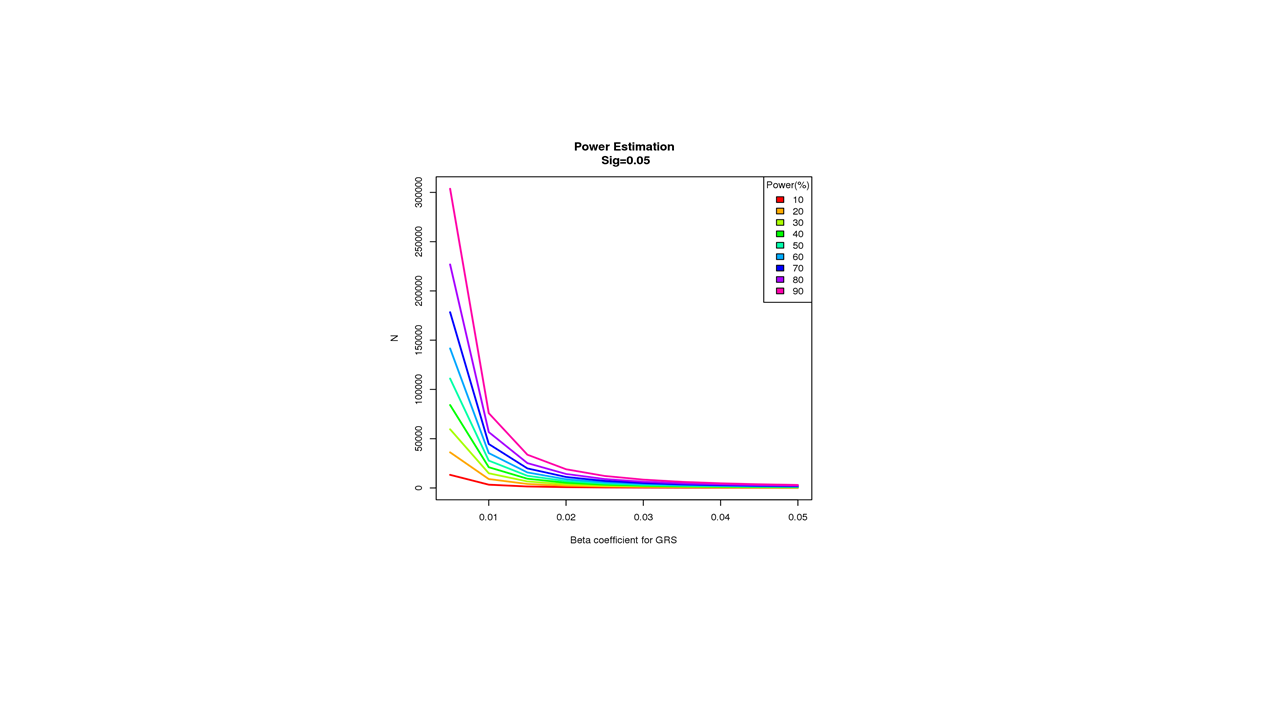

Supplement: S1 Fig — Lines represent the different power values to detect the effect in the model for the association of the weighted genetic risk score (GRS) with the trait. Calculations were performed using Quanto v1.2.4. (TIF) [file pone.0186669.s001.tif]

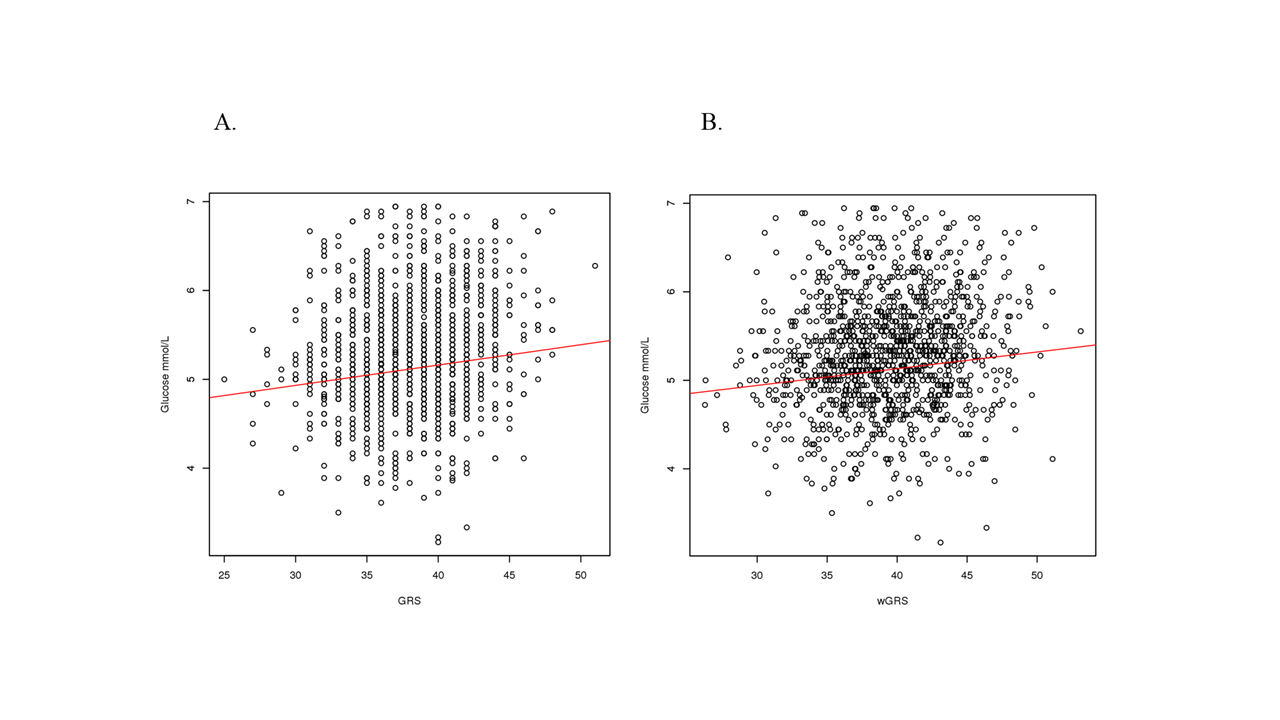

Supplement: S2 Fig — A. unweighted genetic risk score (GRS) and glucose levels (mmol/L) and B. weighted genetic risk score (wGRS) and glucose levels (mmol/L). (TIF) [file pone.0186669.s002.tif]

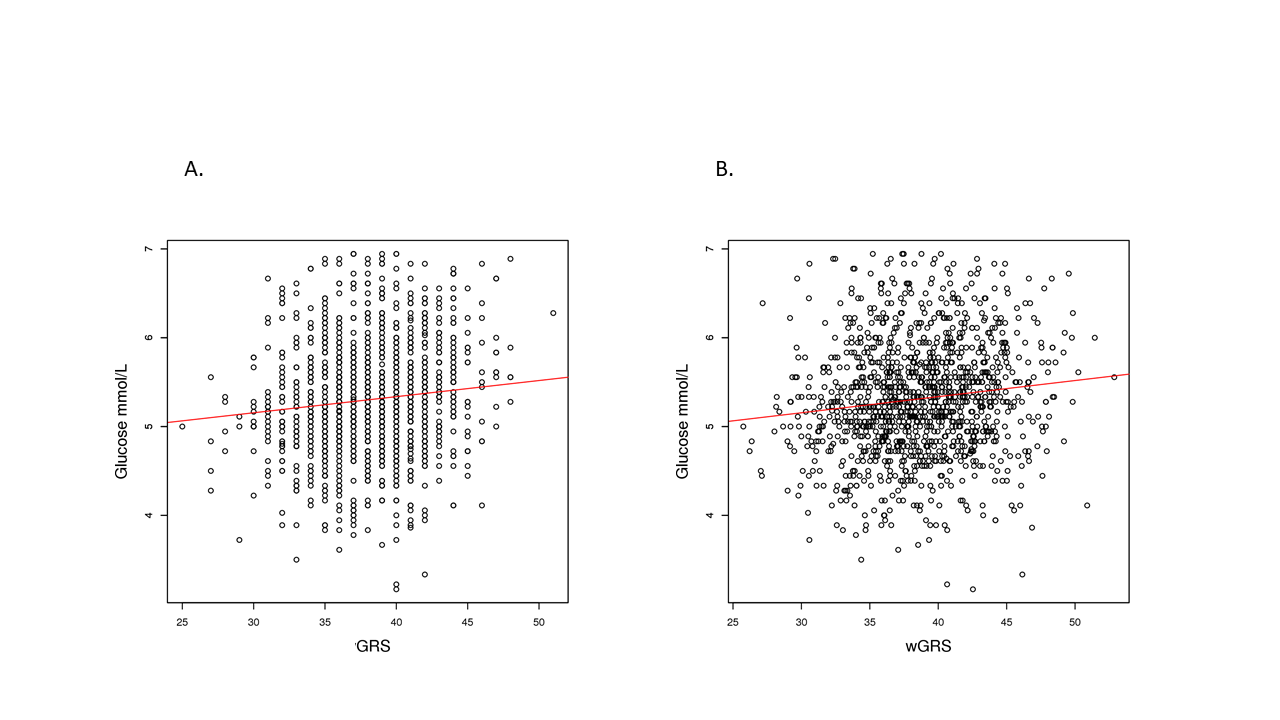

Supplement: S3 Fig — A. unweighted genetic risk score (GRS) and glucose levels (mmol/L) and B. weighted genetic risk score (wGRS) and glucose levels (mmol/L). (TIF) [file pone.0186669.s003.tif]

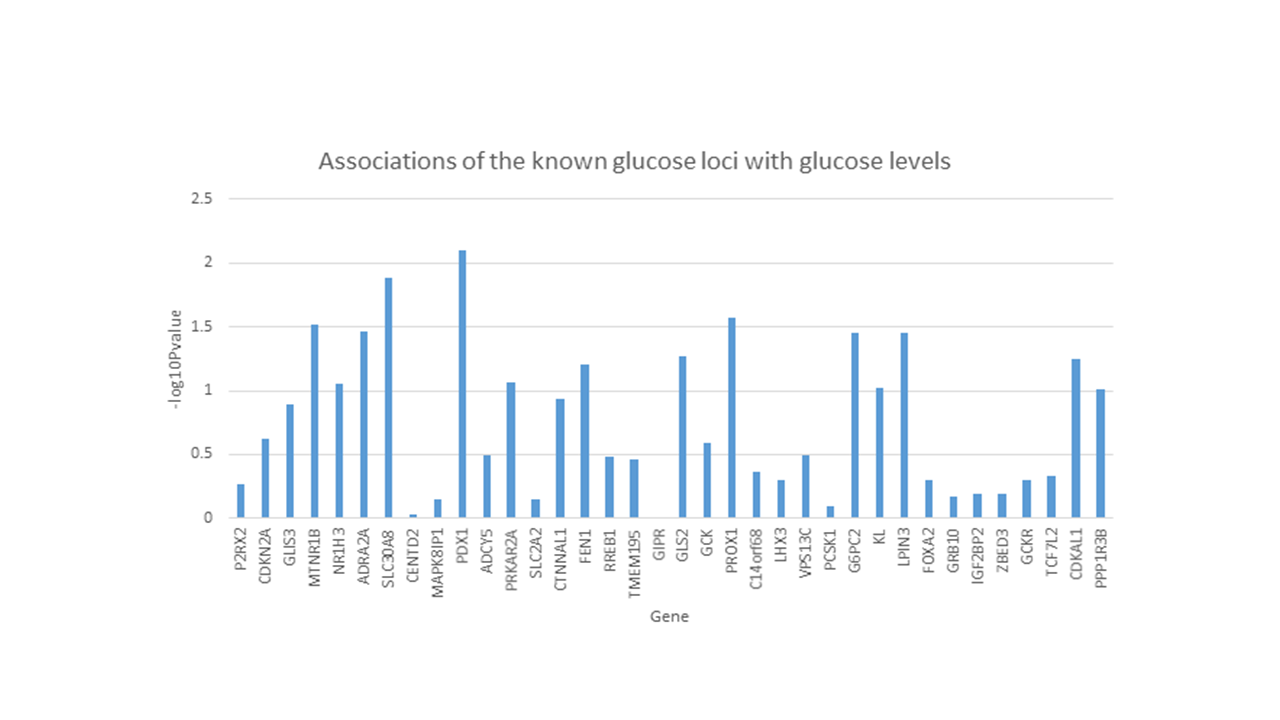

Supplement: S4 Fig — Adjusted for age and sex, x-axis: Gene name, y-axis: -logPvalue. (TIF) [file pone.0186669.s004.tif]
